# CRISPR/Cas9- and Cas3-mediated modification of copy number variation in rice

**DOI:** 10.3389/fgeed.2025.1652950

**Published:** 2025-10-07

**Authors:** Hyungjun Park, Takeshi Kuroha, Hiroaki Saika, Masaharu Kuroda, Hitoshi Yoshida

**Affiliations:** Institute of Agrobiological Science, National Agriculture and Food Research Organization (NARO), Tsukuba, Ibaraki, Japan

**Keywords:** copy number variation, cytosine extension, genome editing, *OsGA20ox1*, phenotype, plant breeding, rice, type-I enzyme

## Abstract

**Introduction:**

Copy number variation (CNV) is one of the crucial elements among genomic structural variations that span plant breeding. However, its impact on agricultural traits has remained elusive.

**Methods:**

We modulated CNVs using two genome-editing technologies, CRISPR/Cas9 and Cas3, along with their verification methods in rice to elucidate the effect of CNVs and further harness to improve relevant agronomic traits.

**Results:**

The addition of cytosine extension to the conventional single-guide RNA and its combination with Cas9 generated a frameshift mutation in parts of the *OsGA20ox1* gene copies, substantially modifying its CNV. Phenotypes of the copy number variants revealed *OsGA20ox1* copy number as a determinant of seedling vigor in rice. The Cas3 nuclease, which induces large-scale deletions, effectively decreased the copy number of the *OsMTD1* gene. We verified the copy number of each gene by combining droplet digital polymerase chain reaction (ddPCR), Sanger sequencing, and bioinformatics tools.

**Discussion:**

Altogether, the two technologies are expected to lay the foundation for new approaches to plant breeding by controlling CNV.

## 1 Introduction

Long-read DNA sequencing technologies have been recently applied to construct high-quality genome sequences and databases and identify structural and copy number variations (CNVs) from multiple perspectives (Fuentes et al., 2019; Qin et al., 2021; Wei et al., 2021; Shang et al., 2022; Wang et al., 2023; Yu et al., 2023). CNV refers to a functional gene with different numbers between cultivars; thus, CNVs represent a form of structural variation denoting specific DNA sequences arising from deletions, duplications, or amplifications. The CNVs have provided insights into plant breeding, including domestication, adaptation, and evolution (Lye and Purugganan, 2019; Wang et al., 2023; Wilson et al., 2025). However, the impact of CNV on agronomic traits remains unclear.

Studies have reported that certain genes, including *OsGA20ox1* and *OsMTD1*, which are possibly associated with agronomic traits, harbor CNVs (Alonge et al., 2020; Liu et al., 2020; Qin et al., 2021; Wang et al., 2023; Wilson et al., 2025). *OsGA20ox1* encodes gibberellin (GA) 20-oxidase, a crucial enzyme involved in the penultimate stage of GA production in rice; it is associated with plant stature as well (Oikawa et al., 2004). This gene is a major quantitative locus affecting the phenotypes such as seedling vigor and grain number per panicle (Abe et al., 2012; Yano et al., 2012; Wu et al., 2016). In addition, it contributes to rice’s tolerance to alkali soil and heat stress and is therefore significant for the next-generation Green Revolution (Guo et al., 2025). *OsGA20ox1* possesses CNVs, particularly in *japonica* rice (Qin et al., 2021; Wang et al., 2023), which have been implicated in several agronomic traits significant for rice breeding. In addition, *OsMTD1*, a gene potentially linked to rice plant architecture and tiller number, has CNVs (Liu et al., 2015; Liu et al., 2020).

Agricultural traits are influenced by a wide range of factors, making it challenging to evaluate the effect of CNVs of a single gene on agronomic traits using cultivars with different genetic backgrounds. The role of CNVs of *OsGA20ox1* on agronomic traits, depending on trait comparison of cultivars with varying genetic backgrounds and overexpression experiments has been previously reported (Wang et al., 2023). Studying agricultural traits and CNVs in the same genetic background can reveal the mechanism by which CNVs influence traits. Establishing NILs with different CNVs is time-consuming, whereas using genome editing for CNV variants with the same genetic background could be beneficial. However, precise modification of CNVs through genome editing remains challenging for the following reasons. Because multiple copies of genes with CNVs arise from the duplication of the genomic region, these copies share mostly identical nucleotide sequences. In particular, a length of 10–20 kb before and after the gene is mostly identical (herein referred to as “gene block”) (Lye and Purugganan, 2019; Qin et al., 2021). This makes it difficult to independently edit one of the duplicated genes using the existing genome editing method with Cas9 nuclease due to its high efficacy and targeted specificity (Wang and Doudna, 2023). Several Cas9 nuclease-independent genome editing technologies have been developed recently (Liu G. et al., 2022). Thus, controlling CNV using different genome-editing technologies is highly anticipated (Shen et al., 2017).

We report technical strategies on two model cases to modify the CNVs and overcome the current limitations of the existing Cas9 nuclease. We modified CNVs by complementing the existing Cas9 method, adding cytosine (Kawamata et al., 2023), and using Cas3 (Dolan et al., 2019; Morisaka et al., 2019), a completely different type of nuclease. Furthermore, we propose genotype detection guides for each approach and effective progeny selection strategies in later generations. We successfully produced dosage-reduced mutant alleles in rice using these strategies. We believe our findings will set practical examples for the effective utilization of the increasingly intensive CNV data from different crops.

## 2 Materials and methods

### 2.1 Target gene selection and CNV identification

A total of 1,003 genes showing CNVs between cv. Nipponbare (NIP) and cv. Koshihikari (KOSH) were extracted from the Rice Resource Center database (https://ricerc.sicau.edu.cn/) (Ricerc, 2019; Qin et al., 2021; Supplementary Table S1). We investigated the function of extracted genes using the Rice Gene Index database (https://riceome.hzau.edu.cn/) (Riceome, 2021; Yu et al., 2023) and existing studies. We selected genes *OsGA20ox1* (Os03g0856700 or LOC_Os03g63970) and *OsMTD1* (Os08g0441300 or LOC_Os08g34249), which are possibly closely related to rice agronomic traits, to confirm the effect of CNV modification in rice (Supplementary Table S2). Next, nucleotide information from each variety was extracted from the Rice Resource Center, annotated, and compared using CLC Main Workbench 25 (Qiagen) and GenomeMatcher to determine the CNV structure of the two genes (Ohtsubo et al., 2008).

### 2.2 Plant materials and culture conditions

*Oryza sativa* L. cv. Nipponbare and cv. Koshihikari were used to generate genome-edited rice. All rice seeds were surface sterilized in 70% ethanol for 5 min and in a 50% sodium hypochlorite solution for 30 min, rinsed with sterile water, placed on Petri dishes with half-strength solid Murashige and Skoog (MS) medium, and subsequently cultured for at least 14 days in a growth chamber. Afterward, the seedlings were moved to soil in black vinyl pots (9 cm in diameter) until they reached maturity. Plants were grown in the greenhouse under LD conditions, which included 10 h of darkness at 25 °C and 14 h of light at 28 °C.

### 2.3 Generation of genome-edited rice

Genome editing was conducted using the vector pZNH2GTRU6 (manuscript in progress) through recognition of the NGG as the PAM sequence. This vector, derived from plasmid pZ2028 (Kuroda et al., 2010), consists of single guide RNAs (sgRNAs) for editing target genes driven by the rice *U6-2* noncoding RNA promoter, *Streptococcus pyogenes* Cas9 driven by the modified rice polyubiquitin promoter, and the hygromycin phosphotransferase (*HPT*) gene driven by the nopaline synthase promoter. The sgRNAs were inserted into the vectors using the *Bbs*I site by ligation.

The vector pZD202-Cas3 (Cas758+Cas1163+hpt) (Saika et al., 2025) was used for genome editing through recognition of AAG as the PAM sequence. This vector contained the *HPT* gene driven by the nopaline synthase promoter, *Cas3* and other Cas genes driven by the modified maize polyubiquitin promoter and CaMV 35S promoter, and CRISPR RNAs (crRNAs) for editing target genes driven by the rice *U6-2* noncoding RNA promoter. The crRNAs were inserted into the vectors using the *Spe*I site by In-Fusion HD cloning (TaKaRa Bio Inc., Shiga, Japan). The vector pOsU6Cas3gRNA rev was used for the crRNA expression vector and is resistant to kanamycin. The crRNAs were inserted into the vectors using the *Bsa*I site by ligation.

The target sequences for sgRNA were selected using CHOPCHOP v3 (Labun et al., 2019) and DeepSpCas9 (Kim et al., 2019). The crRNA sequences were designed manually; sgRNA and crRNA sequences are listed in Supplementary Table S3. Primers and oligonucleotides for construction are listed in Supplementary Table S4.

*Agrobacterium tumefaciens*-mediated transformation was used to introduce transgenes into rice cv. Nipponbare or cv. Koshihikari (Toki, 1997; Ozawa, 2012). Hygromycin-resistant plants were transferred to soil in black plastic pots (9 cm in diameter) until maturity. The genomic DNA was extracted from elongated leaves of all material plants using the Qiagen DNeasy^®^ Plant Mini Kit (Qiagen, Hilden, Germany) to verify genome editing.

### 2.4 PCR, droplet digital PCR (ddPCR), and long-range PCR (LR-PCR)

Polymerase chain reactions (PCRs) were performed with a total volume of 10 μL to investigate the Cas9-induced mutation on *OsGA20ox1* in both Nipponbare and Koshihikari. The PCR mixture contained 5 μL of Emerald Amp^®^ PCR Master Mix (Takara), 1 μL of forward primer, 1 of μL reverse primer, 1 μL of genomic DNA, and 2 μL of betaine. The mixture was thermally cycled in the following conditions: pre-denaturing at 94 °C for 1 min, 40 cycles of PCR at 98 °C for 10 s, annealing at 64 °C for 30 s, and extension at 72 °C for 1 min, and final extension at 72 °C for 5 min, following which the reaction was stopped at 4 °C. The reaction solution was used for direct Sanger sequencing after confirming the band size using gel electrophoresis.

Cycle sequencing PCR was conducted with a total volume of 10 μL using the BigDye^TM^ Terminator v3.1 Cycle Sequencing Kit (Thermo Fisher Scientific, Waltham, MA, United States), following the manufacturer’s instructions. After the PCR, all samples were purified by the ethylenediaminetetraacetic acid (EDTA)/ethanol precipitation method. Next, the pellet was dissolved in Hi-di^TM^ Formamide (Thermo Fisher Scientific) and incubated at 95 °C for 2 min. The reaction solutions were used for the sequence analysis by ABI PRISM^®^ 3130xl genetic analyzer (Thermo Fisher Scientific).

Droplet digital PCR (ddPCR) was used to detect the Cas3-induced CNV change in *OsMTD1* in Nipponbare and the Cas9-mediated CNV change in *OsGA20ox1* in Koshihikari. It was performed in a reaction volume of 22 μL using a QX200 Auto DG Droplet Digital PCR platform (Bio-Rad, Hercules, CA, United States). The PCR mixture contained 11 μL of the ddPCR Supermix for probes with no dUTP, 1 μL of upstream primer, 1 μL of downstream primer, 0.6 μL of the probe, 1.1 μL of HEX (*OsACT1,* PrimePCR Probe Assay: ACT1), 6.3 μL of ddH_2_O, and 1 μL of DNA (20 ng treated with *Hin*d III). The PCR mixture and Droplet generation oil were mixed, and a droplet was generated. The droplet emulsion was thermally cycled in the following conditions: pre-denaturing at 94 °C for 5 min, 40 cycles of PCR at 94 °C for 30 s, and at 55 or 59 °C for 1 min, and melting at 98 °C for 10 min, and finally, the reaction was finished at 4 °C. The completed reaction was used for ddPCR absolute quantification analysis. QuantaLife software was used to determine the nucleic acid copy number. The ratio of the target gene to the reference gene was calculated, and each ratio was converted as a relative value based on the value of the wild-type with one copy as 1.

Next, long-range PCR was performed with a total volume of 20 μL to identify the Cas3-induced deletion size on *OsMTD1* in Nipponbare. The PCR mixture contained 10 μL of KOD One^®^ PCR Master Mix (TOYOBO, Osaka, Japan), 1 μL of the forward primer, 1 μL of the reverse primer, 1 μL of the genomic DNA, and 7 μL of distilled water. The mixture was thermally cycled in the following conditions: pre-denaturation at 94 °C for 5 min, 35 cycles of PCR at 98 °C for 10 s, annealing at 60 °C for 5 s, and extension at 68 °C for 2 min 10 s. The reaction was finished at 4 °C. The reaction solution was used for direct Sanger sequencing after confirming the band size by gel electrophoresis. Primers and probes for all PCR types are listed in Supplementary Table S4.

### 2.5 Genotyping by sanger sequencing and TIDE

The genotyping of genome-edited rice lines of the *OsGA20ox1* gene was confirmed by Sanger sequencing. Sanger sequencing chromatograms were analyzed visually and using the TIDE software (Brinkman et al., 2014) to determine the mutation patterns at each target site (Supplementary Figure S1).

### 2.6 Allelic variant identification via TA cloning and chi-square goodness-of-fit test

Allelic variants were identified to verify the copy number of *OsGA20ox1* PCR products amplified using primers GA20ox1-F, R (Supplementary Table S4) were purified using QIAquick PCR Purification Kit (Qiagen) and subsequently cloned into a pGEM®-T Easy Vector Systems (Promega, WI, United States). In total, 117 independent plasmid clones were selected and sequenced according to the manufacturer’s instructions. The allele ratios of the sequence results were used as the observed values (*O*_*i*_), and the expected allele ratios for each CNV were used as the expected values (*E*_*i*_). A chi-square goodness of fit test (*χ* ^*2*^) was performed to verify the most appropriate CNV.

$$\chi^{2}=\sum \frac{{\left(O_{i}-E_{i}\right)}^{2}}{E_{i}}$$

## 3 Results

### 3.1 Genome editing strategies for CNV modification

Two rice genes (*OsGA20ox1* and *OsMTD1*) and two cultivars (Nipponbare and Koshihikari) were selected as models for CNV modification. Re-annotation was performed after acquiring the nucleotide information of each gene from each cultivar to verify the information of CNVs for these genes from the database and previous studies (Liu et al., 2020; Qin et al., 2021; Wang et al., 2023). In addition, we confirmed the multiplication status of each gene block using PCR analysis following the method described by Liu et al. (2020) to cross-validate sequence data from the database (Supplementary Figure S2).

Re-annotation and sequence comparison revealed that both the genes of interest and the genomic region including the genes (gene blocks) about 13–37 kb were multiplicated. Specifically, the length of the gene block, including *OsGA20ox1,* was approximately 37 kb, and the nucleotide sequences of individual *OsGA20ox1* copies from each block were completely identical. In addition, the nucleotide sequences of gene blocks, including *OsGA20ox1,* shared a similarity of 99.94%. Moreover, gene blocks, including *OsMTD1*, which was 13 kb long, shared completely identical sequences (Supplementary Figure S3; Supplementary Table S5).

**FIGURE 1:**
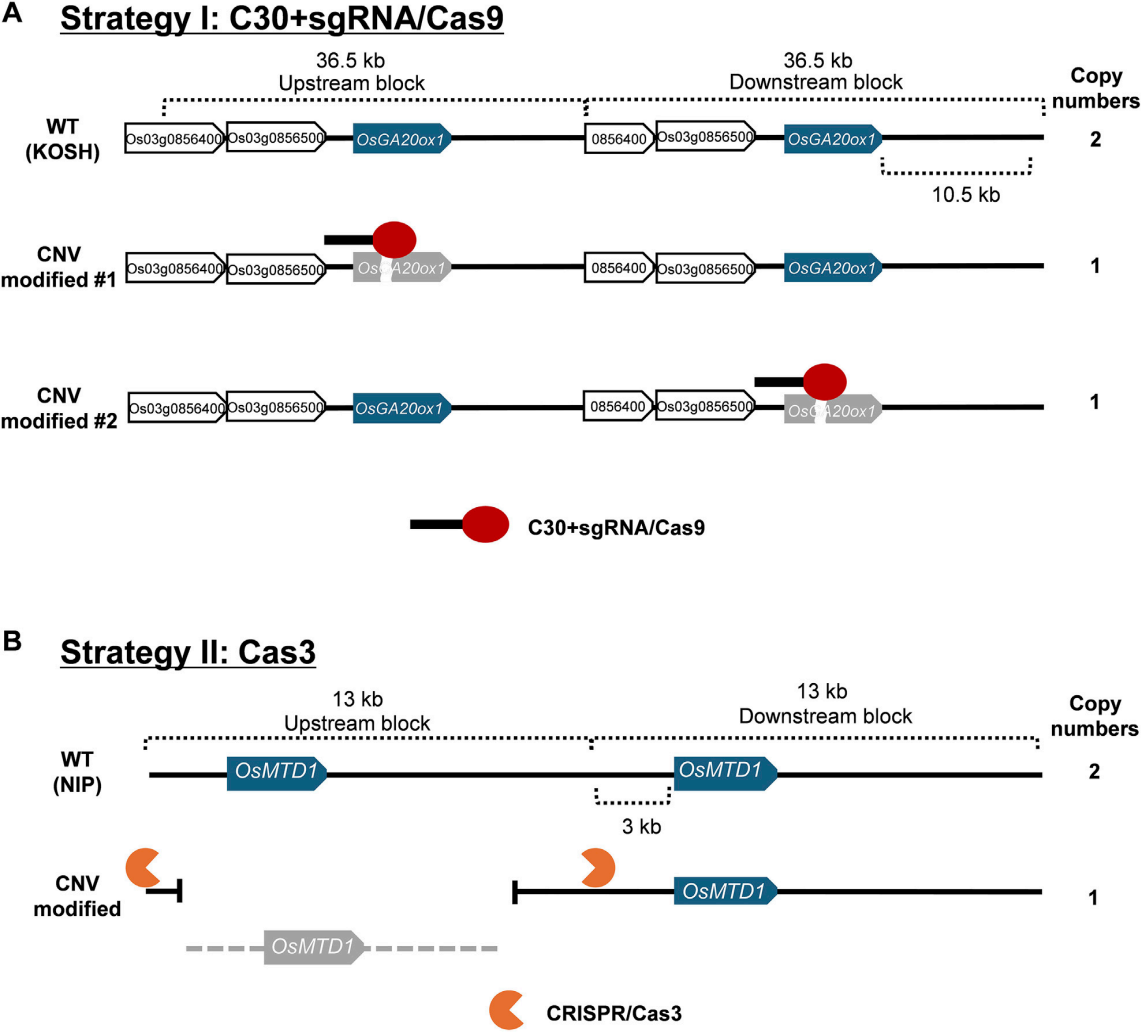
Schematic strategies for genome editing-mediated CNV modification. Information on nucleotide sequences at the expected multiplicated structures was extracted from the Rice Resource Center and analyzed manually. **(A)** Genome editing strategy I uses C30+sgRNA and Cas9 (red ovals with solid black bars). The gene block including *OsGA20ox1* is approximately 36.5 kb in length. Blue boxes indicate the target *OsGA20ox1* genes. White boxes show the other annotated genes in gene blocks. Gray boxes refer to genome-edited dysfunctional genes. **(B)** Genome editing strategy II using CRISPR/Cas3. The gene block including *OsMTD1* is approximately 13 kb in length. Blue boxes indicate the target *OsMTD1* gene. Gray boxes refer to dysfunctional genes due to a large-scale deletion.

**FIGURE 2:**
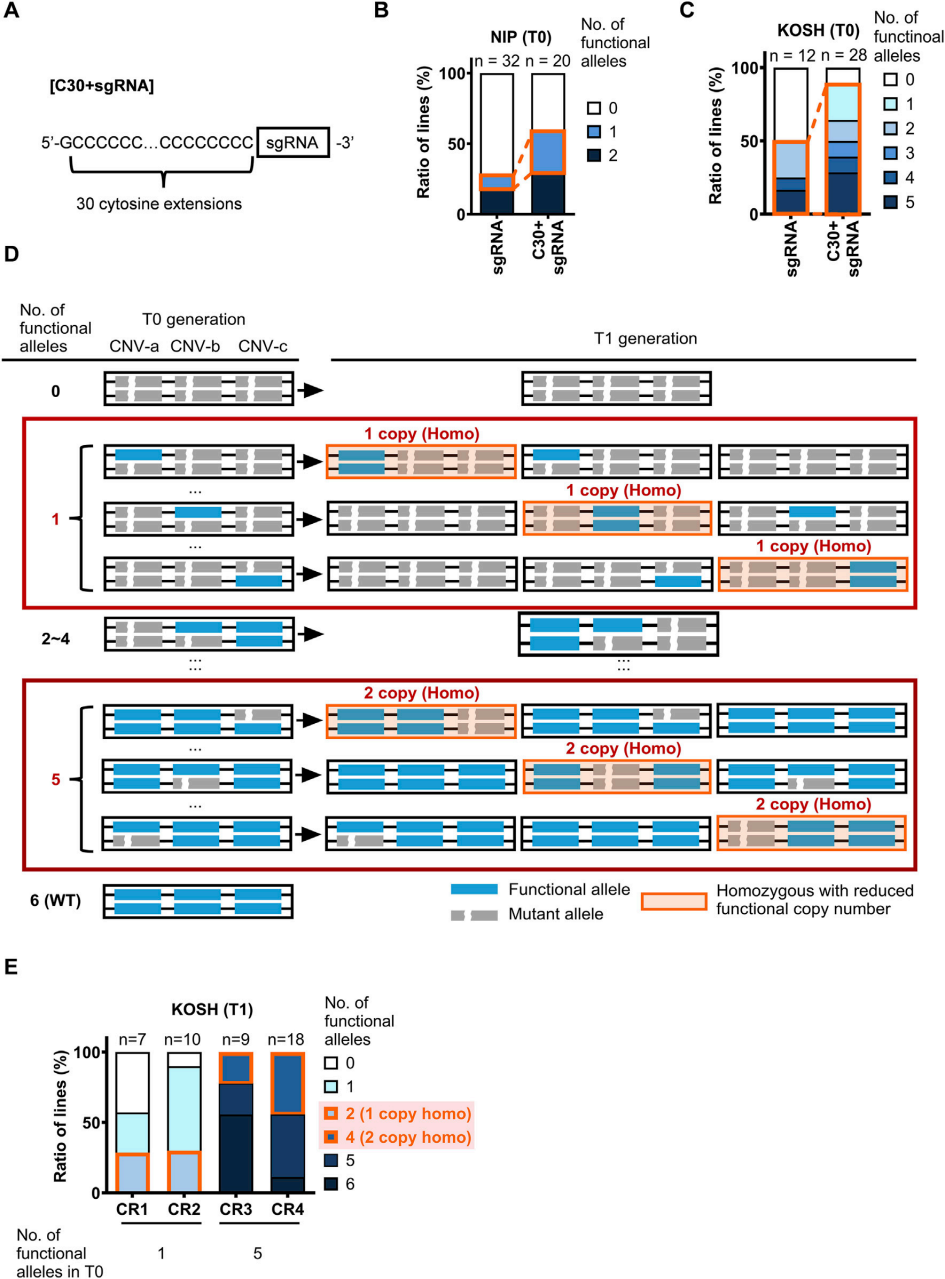
Visualization of genome editing by C30+sgRNA/Cas9. **(A)** Schematic diagram of adding cytosine base extensions to sgRNA. **(B,C)** Comparison of genome-editing efficiency targeted to the *OsGA20ox1* gene using sgRNA and C30+sgRNA in Nipponbare **(B)** and Koshihikari **(C)** T0 generation. Different colors imply the number of functional alleles after genome editing. Orange boxes imply partially modified mutant lines. **(D)** Strategy for efficient selection of progenies with desired copy numbers. The blue box indicates a functional allele. The gray box depicts a mutant allele generated by genome editing. The red rectangles show the efficient region of progeny selection. The orange box indicates the homozygous CNV-modified genotypes. **(E)** Results of progeny selection in Koshihikari T1 generation, according to the selection strategy. Different colors refer to the numbers of functional alleles and genotypes. Orange boxes refer to homozygous lines.

We proposed two strategies for modifying CNVs of these two model genes with substantial nucleotide similarity between gene blocks (Figure 1), i.e., the addition of cytosine extension to sgRNA in CRISPR/Cas9 (Strategy I) and CRISPR/Cas3 (Strategy II). Because the native CRISPR/Cas9 system introduces mutations at high efficiency in rice, most of the genome-edited plants possess bi-allelic mutations for each gene copy (complete knock-outs), making it challenging to obtain partially mutated plants. This implies that native CRISPR/Cas9 could not be efficiently used to obtain genome-edited plants with partially reduced CNVs. Therefore, we employed the first strategy to reduce Cas9 efficiency by adding 30 cytosine bases to the 5′-end of the sgRNA (Figure 2A), because the results of previous studies reported that adding 30 cytosines produced the most dramatic effect (Kawamata et al., 2023). This method affected the concentration of ideal sgRNA-Cas9 complexes and enabled the partial cause of mutations in CNVs by intentionally reducing editing efficiency without affecting target specificity. The second tactic induced large-scale deletions by recruiting Cas3 to the flanking region of the target gene block. Because the deletion orientation of Cas3 is mostly guided from the crRNAs to the PAM (Dolan et al., 2019; Morisaka et al., 2019), we designed crRNAs that caused Cas3-induced deletion toward the target CNV.

### 3.2 Genome editing strategy I: use of sgRNA with 30 cytosine extensions

Genome editing targeting the *OsGA20ox1* exon was performed in Nipponbare to determine whether the sgRNA with 30 additional cytosine nucleotides (designated as “C30+sgRNA”) could effectively reduce genome editing in plants (Figure 2A). Because Nipponbare has one copy of the *OsGA20ox1* gene, and bi-allelic mutations were predominantly induced using the native CRISPR/Cas9 system in rice, we examined whether mono-allelic mutations were induced using the C30+sgRNA by genotyping the T0 generation plants. The genome editing efficiency was reduced when using the C30+sgRNA/Cas9 compared to cases using the normal sgRNA (Figure 2B), which was consistent with the results of a previous study in mammalian cells (Kawamata et al., 2023). Specifically, the proportion of bi-allelic mutant lines decreased from 70% to 40%, and the proportion of mono-allelic and non-mutated lines increased, accordingly.

Next, genome editing with C30+sgRNA/Cas9 was applied to Koshihikari, which contains two copies of the *OsGA20ox1* gene (Qin et al., 2021; Wang et al., 2023), using the same vectors. The genotyping of genome-edited lines revealed that the CR19 line exhibited a segregation pattern of −3, −1, and +1 bp indel mutations in the ratio of 1:2:3, as determined using TIDE analysis (Supplementary Figure S4A). This ratio was unexpected in genomes containing two CNV copies in Koshihikari. In addition, we identified other T0 line harboring more than four mutant alleles (Supplementary Figure S4A). The maximum number of distinct mutant alleles is expected to be four when the CNV is two in Koshihikari, which is inconsistent with the above results. Therefore, the CNV of *OsGA20ox1* was thought to be three or more in Koshihikari. Next, we performed a quantitative analysis using ddPCR to re-examine the copy numbers of *OsGA20ox1* in the Koshihikari and Nipponbare genomes. The relative value was approximateley threefold higher in Koshihikari than in Nipponbare (Supplementary Figure S4B).

For a more accurate verification of the TIDE results, we performed allelic variant identifications using CR19. The target region of *OsGA20ox1* was amplified, and resulting PCR product was cloned. We sequenced 117 independent clones, the proportion of which was consistent with that of TIDE (Supplementary Figure S4C). C in the “+1 insertion” tab in Supplementary Figure S4A was not detected, possibly because of misinterpretation by TIDE. Next, we applied the chi-square goodness-of-fit test to compare multiple CNV hypotheses (CNV = 2, 3, and 4) against the observed allele frequencies derived from cloning and sequencing. The *p-*value (*p* = 0.8568) when the CNV of *OsGA20ox1* was 3 was the most plausible (Supplementary Table S6).

Subsequently, the TIDE results were re-analyzed assuming that Koshihikari had three copies of the target gene, resulting in a consistent interpretation. Similarly, the genome editing efficiency of C30+sgRNA/Cas9 was reduced in Koshihikari (Figure 2C). Compared to the normal sgRNA, the percentage of C30+sgRNA/Cas9-mediated genome editing lines with bi-allelic mutations in all three copies of the target genes significantly decreased from 50% to 10%. Consequently, the diversity of lines with different numbers of functional alleles was increased, indicating the effectiveness of this strategy for modifying CNVs in rice.

### 3.3 Efficient screening of CNV-controlled lines by allele type diversification

The presence of multiple functional allele types complicated the genotyping of subsequent generations in genome editing of genes with more than three copies of CNV. The goal in such a scenario is to obtain progeny lines with one or two CNVs reduced (having two or four functional alleles). Thus, we proposed an effective progeny screening method for such cases (Figure 2D). First, the lines in the middle of the mutation patterns (having two to four functional alleles) were excluded from further screening. Because it is unknown how they will be inherited in future generations, further verification of the next generations is required. The most efficient method to obtain CNV-modified lines is to use lines with one or five functional alleles. These lines can be possibly used to obtain homozygous CNV-modified lines in the next-generation with a theoretical segregation ratio of approximately 25%. We screened T1 according to this strategy and obtained the expected rate (22%–44%) of CNV-modified homozygote lines (Figure 2E).

**FIGURE 3:**
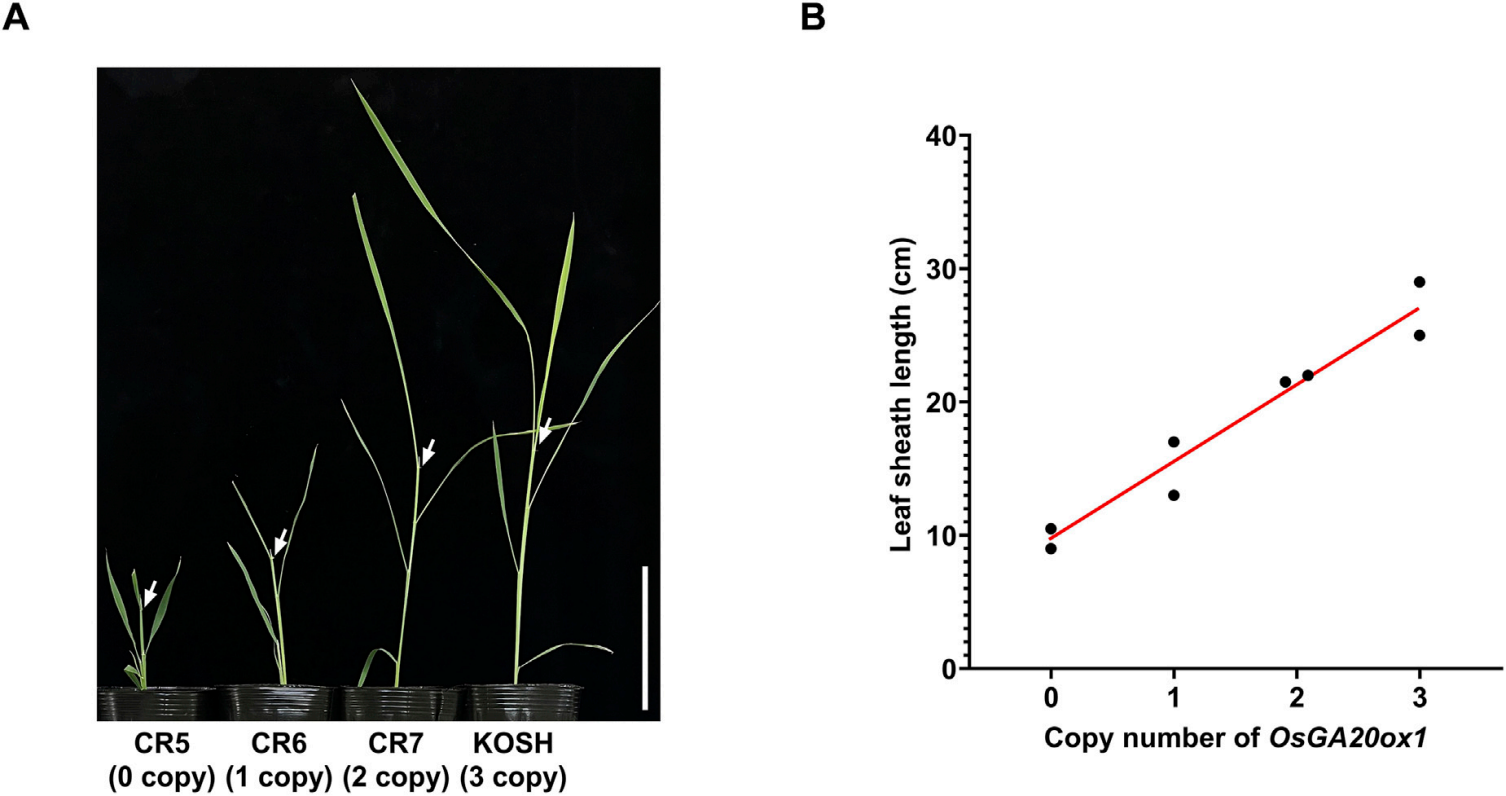
Seedling vigor of *OsGA20ox1* CNV-modified T1 homozygous lines. **(A)** Representative phenotypes of each CNV-modified line 5 weeks after sowing. CR5: All the copies are dysfunctional, CR6: One CNV is functional, CR7: Two CNVs are functional, KOSH: Three CNVs are functional. White arrows refer to the leaf sheath location of each plant. Scale bar (white solid bar) = 10 cm. **(B)** Scatter plot of copy numbers (X-axis) and length of leaf sheath (Y-axis) of each plant. Leaf sheath length was measured 5 weeks after sowing. The red line is a trend line between copy numbers and leaf sheath length.

Next, we compared the seedling vigor of T1 homozygote lines with different copy numbers to examine the effects of copy number differences on *OsGA20ox1*. The leaf sheath length and the copy number of *OsGA20ox1* were positively correlated (Figure 3). Thus, the CNV of *OsGA20ox1* may be a determinant of the seedling vigor in rice. Considering previous reports demonstrating a correlation between the copy number and gene expression (Qin et al., 2021), this phenotype might have been caused by an altered *OsGA20ox1* expression through its CNV modifications.

### 3.4 Establishment of a quantification method for screening CNV-modified lines by mismatching primer

Although TIDE-based genotype evaluation, using Sanger sequencing results, is rapid, cost-effective, and simple, it is susceptible to the amplification pattern of PCR or the accuracy of sequence result wavelength data. Therefore, we established a method to more accurately quantify genome-editing patterns. Because the SpCas9-induced mutation pattern is predominantly limited to small-scale mutations around 3 bp upstream of the PAM sequence, ddPCR technology using mismatch primers was designed. The forward primer was designed to overlap with the DNA cleavage site in the target sequence with an intentional mismatch in the third base from the 3′-end of the primer (Supplementary Figures S5A–E).

**FIGURE 4:**
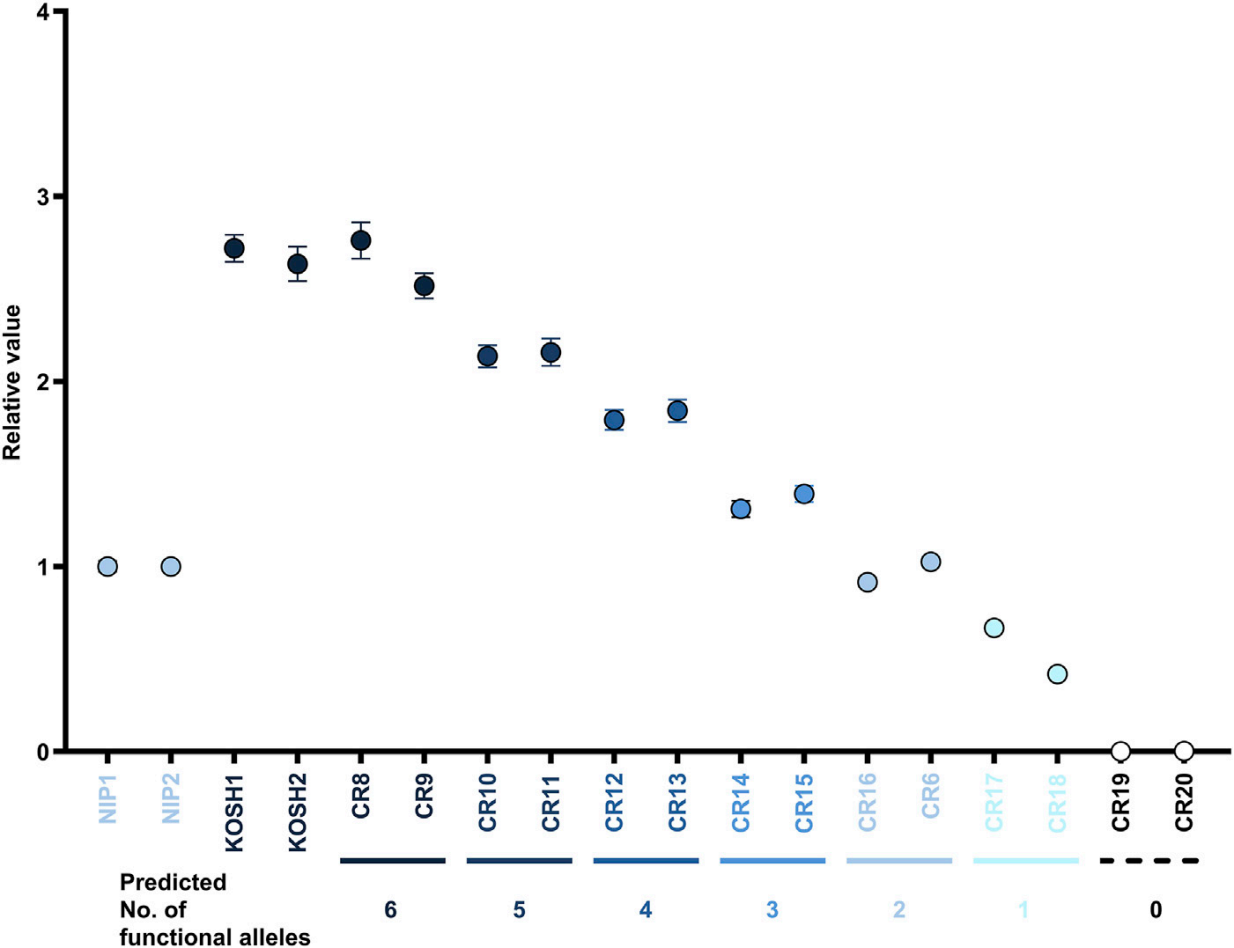
Quantification of unedited functional alleles in C30+sgRNA/Cas9-mediated T0 and T1 genome-edited lines using mismatch primer ddPCR. The expected number of functional alleles by interpretation of TIDE is shown below the X-axis. The Y-axis denotes the relative copy numbers in each sample corresponding to that of NIP. Error bars are calculated by Poisson’s law.

A general PCR conducted before ddPCR revealed no band amplified using the mismatch primers in the 0-functional allele line. A faint band appeared in the low-mutant allele number lines (Supplementary Figure S5F). Each plant with a distinct number of functional alleles, as inferred from the TIDE analysis, was further analyzed using ddPCR. Thus, the stepwise relative value according to each functional allele was detected in ddPCR (Figure 4). The wild-type Koshihikari and T0 plants recovered with six functional alleles in the T1 generation (CR8 and CR9) demonstrated around thrice the relative value of Nipponbare, or T0 plants having two functional alleles (CR16 and CR6). T0 plants having 0-functional alleles (CR19 and CR20) did not react in ddPCR.

### 3.5 Genome editing strategy II: induction of large deletions via Cas3 nuclease

**FIGURE 5:**
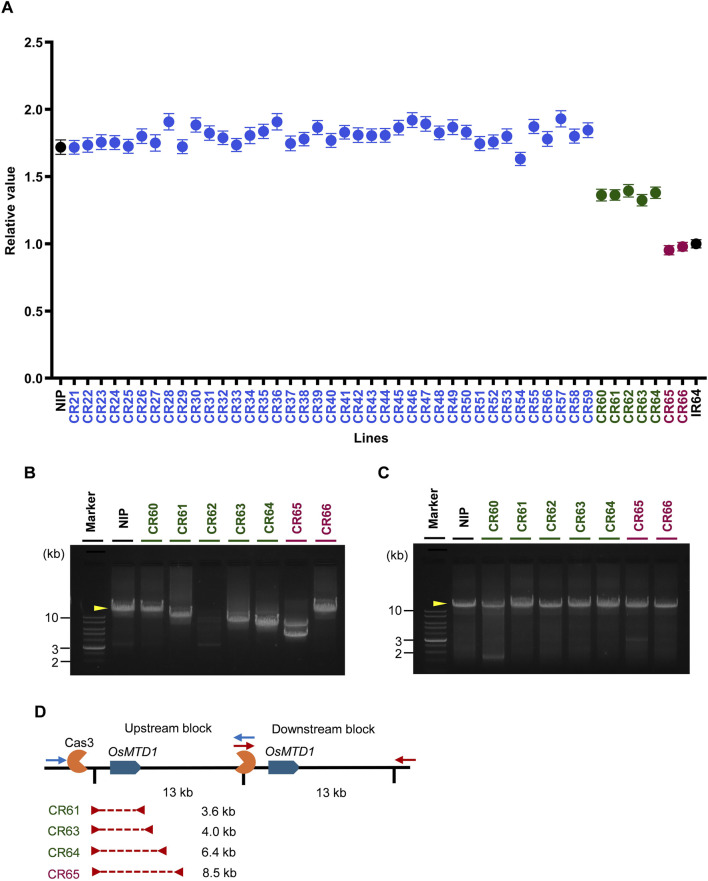
Evaluation of genome editing generated by CRISPR/Cas3. **(A)** Quantification of the numbers of *OsMTD1* alleles using ddPCR in Cas3-introduced Nipponbare lines at T0 generation. The X-axis shows sample names. The Y-axis denotes the relative values of each sample corresponding to those of IR64. Error bars are calculated by the Poisson’s law. Black: Nipponbare (two copies) and IR64 (one copy). Blue: No genome editing occurred, or deletions did not reach the probe position in the *OsMTD1* region. Green: CNV was modified heterozygously. Purple: CNV was modified homozygously. **(B)** Gel electrophoresis analysis of the *OsMTD1* upstream block in CNV-modified T0 lines. Yellow arrowhead, fragment size amplified in wild-type. **(C)** Gel electrophoresis analysis of *OsMTD1* downstream block in CNV-modified T0 lines. Yellow arrowhead, fragment size amplified in wild-type. **(D)** Schematic diagram showing the deletion size in the *OsMTD1* CNV-modified T0 lines. Blue boxes indicate the target *OsMTD1* genes. Blue and red arrows denote long-range PCR primers for upstream and downstream blocks. The Red dashed line refers to the deletion site.

Next, we wanted to determine whether inducing Cas3 nuclease-mediated large-scale deletions would be effective in modifying CNV, we designed experiments in which a single sequence on the outside of a gene block was used to induce deletions of the upstream block inward (Figure 1B). Cas3-mediated genome editing was conducted on Nipponbare, which has two copies of the target gene *OsMTD1*, and CNV was surveyed using ddPCR. Nipponbare and IR64 cultivars were used as positive controls with two copies and one copy of *OsMTD1*, respectively. Among the 46 T0 generation lines, seven lines, accounted for 15.2%, demonstrating a decreased relative value of the target gene compared with Nipponbare (Figure 5A). The ddPCR results demonstrated that the T0 generation was divided into three groups containing 1. no deletions reaching the *OsMTD1* region or no genome editing occurrence (blue), 2. heterozygous deletions occurrence (green), and 3. homozygous deletion occurrence (purple).

Next, the deletion size was investigated by long-range PCR in each T0 CNV-modified line. We first conducted long-range PCR to detect the deletions in the *OsMTD1* upstream block (Figure 5B). We could not detect deletion at least on gel electrophoresis in lines CR60, CR62, and CR66 because of almost the same size of PCR products (CR60 and CR66) or no amplification (CR62). In contrast, a decrease in the PCR product size was detected in lines CR61, CR63, CR64, and CR65 on gel electrophoresis. No deletion was observed in the *OsMTD1* downstream block of all lines (Figure 5C). Sanger sequencing of PCR products revealed that CR61 and CR63 had 3.6 and 4.0 kb deletions that barely reached the *OsMTD1* region in the upstream block. CR64 had a slightly longer deletion of 6.4 kb, whereas CR65 had the longest deletion of 8.5 kb (Figure 5D).

**FIGURE 6:**
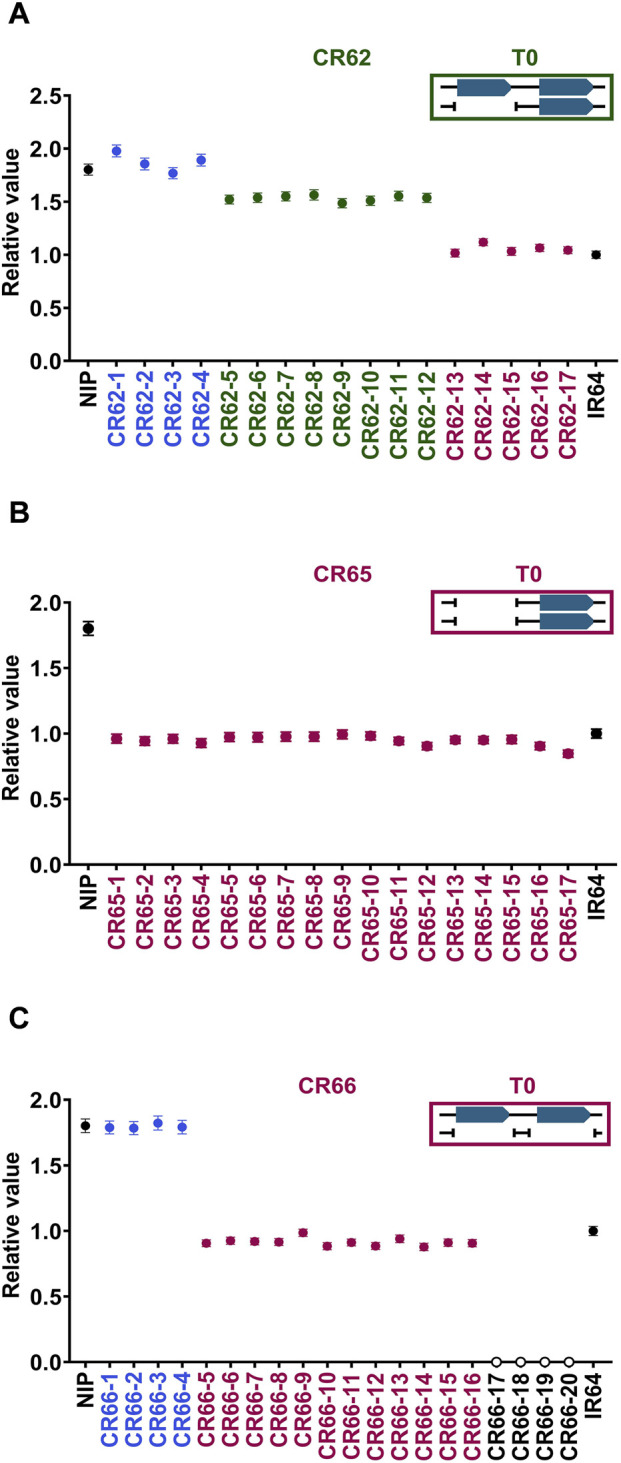
Segregation patterns of *OsMTD1* CNV-modified T1 individuals analyzed by ddPCR. Segregation of T1 progenies of CR62 **(A)**, CR65 **(B)**, and CR66 **(C)** T0 lines, respectively. Black: Nipponbare (two copies) and IR64 (one copy). Blue: no genome editing occurred, or deletions did not reach the probe position in the *OsMTD1* region. Green: CNVs were modified heterozygously. Purple: CNVs were modified homozygously. Yellow: Two CNV sites were deleted homozygously. Boxes in the upper right are schematic diagrams of the expected genotypes of each line at T0 generation.

Subsequently, T1 generation lines presumed to have a decreased CNV in the T0 generation were genotyped to confirm the homozygosity. First, CR62, one of the heterozygous (green) T0 groups, was segregated into three groups in the next-generation; we obtained approximately 30% of the CNV-modified lines (purple) (Figure 6A). The target band was not observed in the LR-PCR results from the CR62 T1 individuals (Supplementary Figure S6), indicating that the binding site of the forward primer was deleted (Supplementary Figure S6B). Next, CR65, a CNV-decreased homozygous T0 group (purple), was confirmed to have a fixed CNV-decreased homozygous genotype in all the lines (Figure 6B). Finally, we could not obtain CNV-modified homozygous lines with the desired number of CNV (CNV = 1) for CR66, which was another CNV-decreased homozygous group (purple), unlike CR65 (Figure 6C). Taken together, homozygous genome-edited lines with desired CNVs can be selected using ddPCR-based genotyping of T1-segregating individuals. No clear phenotypic changes were observed in the *OsMTD1* CNV modified lines of the T0 and T1 generations, including the homozygous knockout lines (CR66-17/18/19/20).

## 4 Discussion

Despite the growing interest in CNVs, the mechanism controlling their numbers has rarely been studied (Li et al., 2017; Shen et al., 2017). We demonstrated that CNV modification of each target gene, especially by partial reduction of functional gene copies, can be successfully achieved with genome editing technologies. We proposed two independent strategies, i.e., I. controlling Cas9 activity by adding 30 cytosine extensions to sgRNA, and II. inducing large-scale deletions using Cas3 nuclease. The findings provide significant insights into future research directions for modulating and optimizing CNV through genome editing.

### 4.1 CNV modification using emerging genome-editing technologies

The SpCas9 in rice displays high activity, predominantly causing bi-allelic mutations rather than mono-allelic mutations for each target gene. This is also the case for other plants (Li et al., 2018; Naim et al., 2018; Wolabu et al., 2020; Lu and Tian, 2022; Yuan et al., 2024). Because several CNVs are caused by the multiplication of genome regions including the numbers of genes with almost identical nucleotide sequences, obtaining genome-edited plants with a partially reduced copy number of the target gene is challenging. This problem was solved by reducing and optimizing the activity of Cas9. Kawamata et al. (2023) reported that the addition of cytosine between 15 and 30 to the 5′-end of sgRNA caused mono-allelic mutations, instead of bi-allelic mutations, in animal cells. We demonstrated the effect of controlling the genome-editing frequency of Cas9 by adding 30 cytosines to sgRNA in plants, and its application to modify CNV in rice. This technique would be possible in other plants as well. Moreover, such mosaic editing may be useful in studying genes causing lethality or sterility as recessive homozygous (bi-allelic) mutations. A previous report on rice suggested that editing efficiency can be controlled by adjusting the number of nucleotides in the sgRNA, and this approach is also expected to facilitate the efficient modification of CNV (Liu X. J. et al., 2022).

Cas3 is characterized by easy induction of large-scale deletions compared to Cas9 (Dolan et al., 2019; Morisaka et al., 2019). We successfully modified CNV using Cas3 in the second strategy, targeting *OsMTD1*, which has a 13 kb gene block (Figure 5). Although it did not delete the entire 13 kb gene block, the ddPCR experiment demonstrated a reduced copy number of *OsMTD1*, being a good example for modulating the CNV of the target gene. Approximately 15% of the Cas3-mediated genome-edited plants displayed altered CNVs (Figure 5A), which was similar to the efficiency obtained previously (Li et al., 2023). This appears to be sufficient efficiency to obtain enough numbers of CNV-modified lines.

**FIGURE 7:**
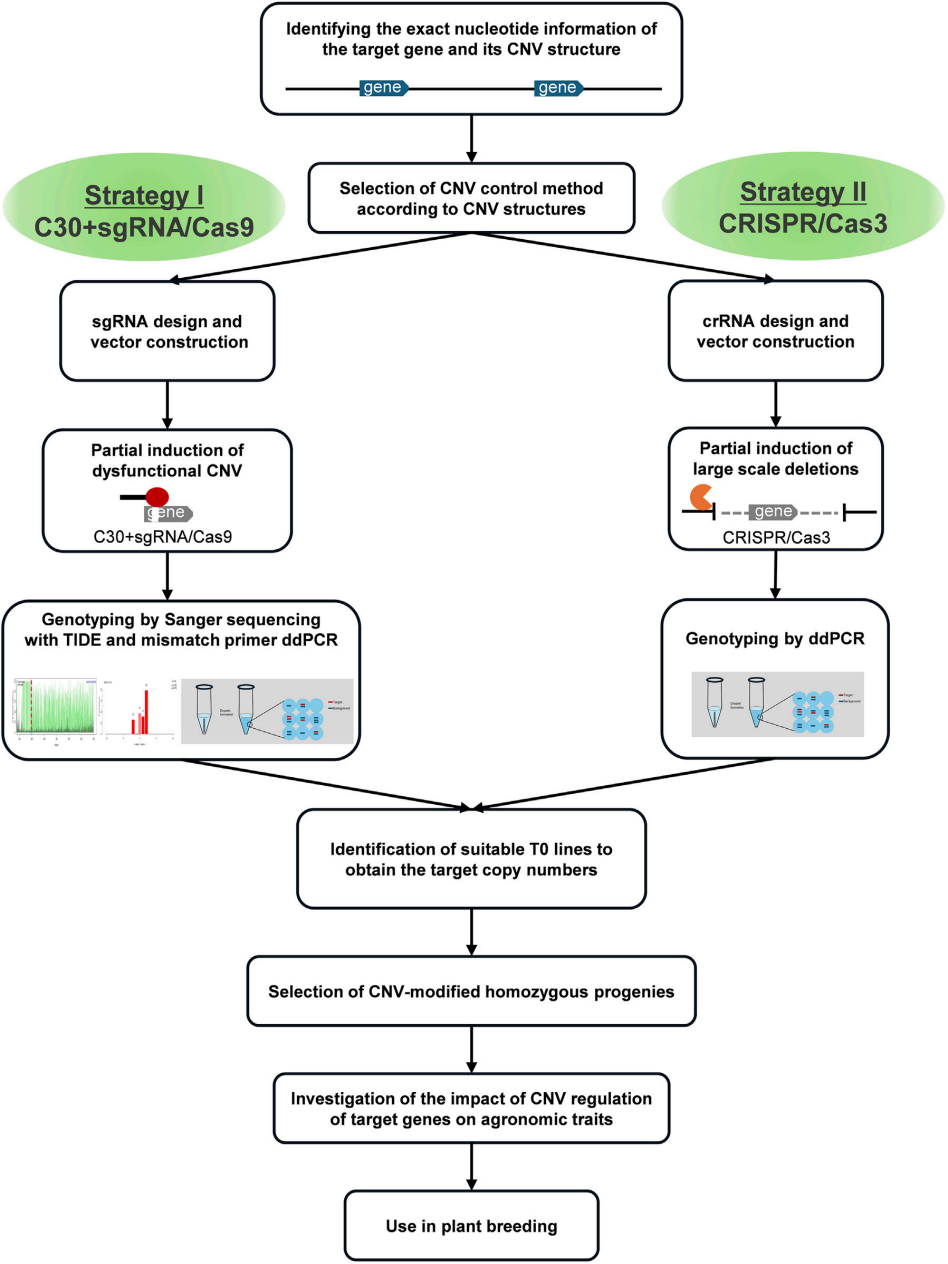
Flowchart of genome editing-mediated CNV modification in plant breeding.

Each strategy has its advantages and limitations in several aspects (Table 1). An appropriate strategy depending on the purpose of the study and the structure of the CNV of the target gene should be adopted (Figure 7).

**TABLE 1:** Advantages and limitations of each strategy.

	Strategy I: C30+sgRNA/Cas9	Strategy II: Cas3
Specificity	Targeting a specific copy is impossible	Targeting a specific copy is possible
Specificity	Editing only a single exon of the target gene	Editing the area surrounding the target gene
Efficiency	Not affected by the length of the gene block	As the length of the gene block increases, the efficiency decreases
Target sequence/PAM	Possibility of variation in efficiency due to sgRNA	The design of the PAM sequence is limited
Genotyping method	TIDE, ddPCR	ddPCR, long read sequencing
Cost	Relatively low	Relatively high

### 4.2 Copy number evaluation in genome-edited plants

Because ddPCR is a useful technique to determine the copy number of target genes (Collier et al., 2017), we used it to evaluate the CNVs of genome-edited lines. We quantified the copy number of the target gene in Cas3-mediated genome-edited plants compared to that in the wild-type.

We successfully applied the TIDE software to evaluate the mutation pattern and CNV caused by the C30+sgRNA/Cas9 system (Supplementary Figures S1, S4A). Despite being an intuitive, rapid, and cost-effective software, TIDE has the drawback that the quality of the sequence reads and PCR product purity determine the reliability of the results (Brinkman et al., 2014). A previous study measured the efficiency of Cas9-induced genome editing using ddPCR (Peng et al., 2020). We developed a ddPCR method with a deliberately mismatched primer that could more accurately verify multiple copy numbers in genome-edited plants generated by the C30+sgRNA/Cas9 system (Figure 4; Supplementary Figure S5). Primers utilizing intentional mismatches have been successfully used for gene or cultivar screening using the SNP data (Hayashi et al., 2004; Park et al., 2020). A single base mismatch at the 3′ end is usually insufficient for accurate discrimination, and intentional addition of the mismatch in the PCR primer can resolve this problem (Hayashi et al., 2004). Because the predominant sites of Cas9-induced mutations are limited, and the actual mutation patterns can be identified by Sanger sequencing, we could distinguish the wild-type and edited mutant alleles by using such mismatch primers (Figure 4; Supplementary Figure S5). Regarding the stability of this technique, even for the same samples or lines assumed to have the same number of functional alleles, slight fluctuations in the ddPCR values were occasionally observed, as previously reported (Collier et al., 2017). Therefore, cross-validation of two methods, TIDE and ddPCR using mismatch primer, is recommended to accurately determine reduced CNV.

### 4.3 Impact of CNV modification on agronomic traits

The genotyping and ddPCR results demonstrated that Koshihikari may have three copies of the *OsGA20ox1* gene, rather than two. These results are inconsistent with those of previous studies reporting a copy number of two of *OsGA20ox1* in Koshihikari (Qin et al., 2021; Wang et al., 2023). Moreover, the phenotypes of CNV-modified lines support the hypothesis that Koshihikari possesses three copies of *OsGA20ox1* (Figure 3). Such a misestimation of CNV has been reported in *Arabidopsis* using a probe-based technique, which differs from the results of previous whole-genome sequencing study (Samelak-Czajka et al., 2017). These inconsistencies may be due to technical limitations, accumulation of genomic data, interpretation errors due to computational interpretation bias, and genetic complexity. Therefore, cross-analysis using different types of experimental techniques, such as probe- or sequence-based techniques, is necessary to correctly identify CNVs. We comprehensively utilized three analytical techniques in this study. First, using TIDE, a software that interprets data based on sequencing wavelengths, we found that the proportion of alleles did not match existing data. Second, we used two different probes in ddPCR technology, which enabled absolute quantification. Finally, we isolated genetic fragments from CR19 and performed Sanger sequencing and statistical analyses, demonstrating that three CNVs were the most persuasive.

Previous studies reported that the expression of *OsGA20ox1* controls the seedling vigor in rice; however, the genetic basis for its function has been unclear (Oikawa et al., 2004; Abe et al., 2012; Yano et al., 2012). The leaf sheath length of CNV-modified lines was measured to evaluate the regulatory roles for CNV of *OsGA20ox1* in agronomic traits. Our results demonstrated stepwise differences in leaf sheath length depended on the copy number of *OsGA20ox1* (Figure 3), and supported the idea that the difference in CNVs, not SNPs, of *OsGA20ox1* affected the seedling vigor. In addition, the change in the expression of *SlKLUH*, a major gene related to increased tomato fruit weight, was previously assumed to be caused by a nucleotide alteration in the promoter region. However, Alonge et al. (2020) revealed that the CNV of *SlKLUH* is the direct cause of the change in the expression and agronomic traits. These findings are consistent with those of previous reports that the gene expression change depends on differences in CNV (Chiang et al., 2017; Lye and Purugganan, 2019; Qin et al., 2021; Wang et al., 2023).

Several recent studies reported that both simple functional knockout of target genes and fine-tuning of the gene expression through genome editing of *cis*-regulatory regions would be promising approaches to developing applicable genome-edited crops (Rodriguez-Leal et al., 2017; Wang et al., 2021; Ludwig et al., 2023; Zhou et al., 2024; Kuroha et al., 2025; Lanctot et al., 2025; Mou et al., 2025). Because the *OsGA20ox1* is a crucial gene associated with several agronomic traits in rice, fine-tuning of this gene through CNV modification may have the potential to optimize appropriate seedling vigor and plant height, such as the *SD1*/*OsGA20ox2* gene (Monna et al., 2002; Sasaki et al., 2002). In addition, our results suggest that fine-tuning of CNVs in agronomically important genes can contribute to the genetic improvement of multiple crops, including polyploid species.

### 4.4 Application of genome-editing-mediated CNV modification in breeding

We proposed a strategy to efficiently screen CNV-modified lines (Figure 2D) and successfully obtain CNV-modified homozygous lines at T1 generation (Figure 2E). This strategy could be applied to cases even when we aim to obtain lines with the desired copy number of the gene whose CNV is more than three. We designed a model for the increased copy number of the target gene to determine the number of functional alleles in T0 individuals suitable for the efficient selection of the progenies with the desired number of copies (Table 2). Here, we defined “the number of functional alleles at T0 generation that was suitable for efficient selection of progenies with desired CNV” as “functional allele numbers for selection (FAS)”. Next, based on the designed model, we proposed a formula to easily calculate FAS as a function of the number of desired gene copies “x.”

$$FAS\left(x\right)=2x\pm 1,\;\;\;0<x<n$$

**TABLE 2:** Example of an efficient number of functional alleles to obtain homozygous lines in T1 generation when CNV is more than three.

Number of CNVs in the wild-type genome (*n*)	Desired number of CNVs (x)	Functional allele numbers for selection (FAS (x))	Number of CNVs in definitive homozygous T1
*n* = 2	x = 1	FAS (1) = 1 or 3	1
*n* = 3	x = 2, 1	FAS (1) = 1 or 3* FAS (2) = 3* or 5	1 2
…	…	…	…
n=∞	x = n-1, n-2, … 3, 2, 1	FAS (1) = 1 or 3* FAS (2) = 3 or 5 FAS (3) = 5 or 7 … FAS (n-2) = 2n-3 or 2n-5 FAS (n-1) = 2n-1 or 2n-3*	1 2 3 … n-2 n-1

*Refer to items at both ends of the FAS, overlapping with the adjacent values and were excluded.

Finally, as depicted in Table 3, we simulated the usefulness of the FAS in CNV-modified line screening. We assumed that the CNV of the wild-type genome was eight, the desired CNV was five, and FAS (5) was calculated as 9 or 11. Thus, we estimated that the probability of obtaining a homozygous line with the target copy number (five) in the T1 generation was the highest (17.1%–18.0%) when the two values (9 or 11) of FAS obtained through the formula were taken. They were about thrice higher than the probability calculated in not FAS (7 or 13). Homozygous genome-edited progenies with the desired CNV could be selected following this strategy, using a variety of edited allele types of T0 individuals.

**TABLE 3:** Simulation example when the total CNV was eight and the desired number of copies was five.

Simulation example [*n* = 8, x = 5] → FAS (5) = 9 or 11			
Number of total functional alleles	Number of functional alleles on each chromosome in T0	Percentage of each genotype in T0 (%)	Probability of homozygous lines acquisition from all T1 seeds (%)
7	7/0 or 0/7	0.2	-
	6/1 or 1/6	3.9	-
	**5/2 or 2/5**	**27.4**	**6.9**
	4/3 or 3/4	68.5	-
**9**	8/1 or 1/8	0.2	-
	7/2 or 2/7	3.9	-
	6/3 or 3/6	27.4	-
	**5/4 or 4/5**	**68.5**	**17.1**
**11**	8/3 or 3/8	2.6	-
	7/4 or 4/7	25.6	-
	**6/5 or 5/6**	**71.8**	**18.0**
13	**8/5 or 5/8**	**20.0**	**5.0**
	7/6 or 6/7	80.0	-

The cases in which the total number of functional alleles was 7, 9, 11, and 13 in the T0 generation are shown. Red bold letters emphasized the number of cases in which the homozygous lines with desired copy numbers were obtained in the case of FAS. The black bold letters indicate the number of cases in which the homozygous lines with the desired copy numbers were obtained in the case of not FAS.

In conclusion, we successfully modulated CNV using two technologies, CRISPR/Cas9 and Cas3, in both model cases. Our approach provides a solid foundation for future application of these techniques in agriculture by modifying CNVs related to agronomic traits. This strategy may not only reinforce the effectiveness of genetic modifications in different crops but also open new avenues for optimizing plant breeding for several purposes.

## Data Availability

The datasets presented in this article are not readily available because No restriction. Requests to access the datasets should be directed to Hitoshi Yoshida, yoshida.hitoshi920@naro.go.jp.
