# Supplementary material for: CRISPR/Cas9- and Cas3-mediated modification of copy number variation in rice
Source: Front Genome Ed. 2025 Oct 7;7:1652950. doi: 10.3389/fgeed.2025.1652950 (PMC12537685; doi:10.3389/fgeed.2025.1652950)
Supplement: Supplementary file 2 [file Supplementaryfile1.docx]

Supplementary Material

# Supplementary Figures

**Supplementary Figure 1.** Examples of Sanger sequencing (upper) and TIDE analysis (bottom) of *OsGA20ox1* genome-edited lines in Nipponbare. Red and blue boxes indicate the target sequence and the PAM site, respectively. The TIDE analysis provides a ratio (Y-axis) for each InDel size (X-axis). The light red bar and red bars represent the ratio of a functional allele (WT sequence) and mutant alleles, respectively. The *R*^2^ and *p*-values indicate a goodness-of-fit measure and the statistical significance for each InDel was calculated. The total eff. denotes the efficacy of genome editing.

**Supplementary Figure 2.** PCR-based confirmation of CNVs for *OsGA20ox1* and *OsMTD1*. (A) Schematic model of the gene blocks, including the target gene. Arrows indicate the positions of PCR primers for verifying the tandem duplication of gene blocks. Primer pairs A/B, B/C, and C/D were used for analyzing tandem duplication events. (B) PCR analysis of the CNV region including *OsGA20ox1* in Koshihikari and Nipponbare. The results demonstrate that Koshihikari and Nipponbare possess more than two and a single copy of the gene block, respectively. Arrowhead, predicted fragment size amplified when each junction structures exist. (C) PCR analysis of the CNV region including *OsMTD1* in Nipponbare and IR64. The results demonstrate that Nipponbare and IR64 possess more than two and a single copy of the gene block, respectively. Arrowhead, predicted fragment size amplified when each junction structures exist.

**Supplementary Figure 3.** Comparison of nucleotide sequences between gene blocks in the CNV regions. (A) Comparison of *OsGA20ox1* DNA sequences between CNVs. Red boxes indicate CDS sequences. (B) Comparison of *OsGA20ox1* gene block DNA sequences of Koshihikari. Green boxes denote other annotated genes. Red boxes indicate the *OsGA20ox1* location. (C) Comparison of the *OsMTD1* gene block DNA sequences of Nipponbare. Red boxes indicate the OsMTD1 location. One square in the grid means 1 kb.

**Supplementary Figure 4.** Copy number estimation of *OsGA20ox1* in Koshihikari. (A) Example of TIDE analysis of genome-edited line CR19 and CR67 in Koshihikari. The TIDE analysis provides a ratio (Y-axis) for each InDel size (X-axis). The red bars represent the ratio of mutant alleles. The bar graphs in the black box represent the ratio of each A, T, G, and C nucleotide for the case of +1 insertion. (B) ddPCR results of Nipponbare and Koshihikari. The X-axis shows sample names. The Y-axis denotes the relative values of each sample corresponding to that of Nipponbare. The Poisson law was used to calculate error bars. (C) Allelic variant identification results of CR19. The red characters denote mutations. The bold characters point out PAM sequences.

**Supplementary Figure 5.** Design of mismatch primers for evaluating the mutant allele frequency with ddPCR. (A) Schematic diagram of the *OsGA20ox1* target site in our ddPCR design. Green nucleotide refers to target sequences. Blue nucleotide refers to PAM sequences. Blue and black arrows refer to each forward and reverse primer. The red arrow indicates the DNA cleavage site. The green star denotes the mismatched nucleotide site. Minor groove binder (MGB) is a Tm enhancer. (B) Case example of T insertion, two mismatches made no PCR products. The red background indicates an insertion nucleotide. Gray backgrounds denote a mismatched nucleotide. (C) Expected number of mismatches when designing a mismatch primer at the DNA cleavage site A to G. (D) Expected number of mismatches when designing a mismatch primer at the DNA cleavage site A to T. (E) Expected number of mismatches when designing a mismatch primer at the DNA cleavage site A to C. (F) Validity analysis on the primer detecting polymorphisms between genome-edited lines in Koshihikari. The arrowhead indicates an amplified target fragment.

**Supplementary Figure 6.** Analysis of a rare deletion pattern in the CR62 line. (A) Gel electrophoresis analysis of the *OsMTD1* upstream block in CNV-modified CR62 T1 lines. Yellow arrowhead, fragment size amplified in wild-type. (B) Schematic diagram showing the expected deletion pattern in the *OsMTD1* CNV-modified T0 CR62 line. Blue boxes indicate the target *OsMTD1* genes. Blue and red arrows denote long-range PCR primers for upstream and downstream blocks, respectively. The red dashed lines refer to the deletion sites on each chromosome (CR62-A and CR62-B).
