# Supplementary figures and images for: CRISPR/Cas9- and Cas3-mediated modification of copy number variation in rice
Source: Front Genome Ed. 2025 Oct 7;7:1652950. doi: 10.3389/fgeed.2025.1652950 (PMC12537685; doi:10.3389/fgeed.2025.1652950)

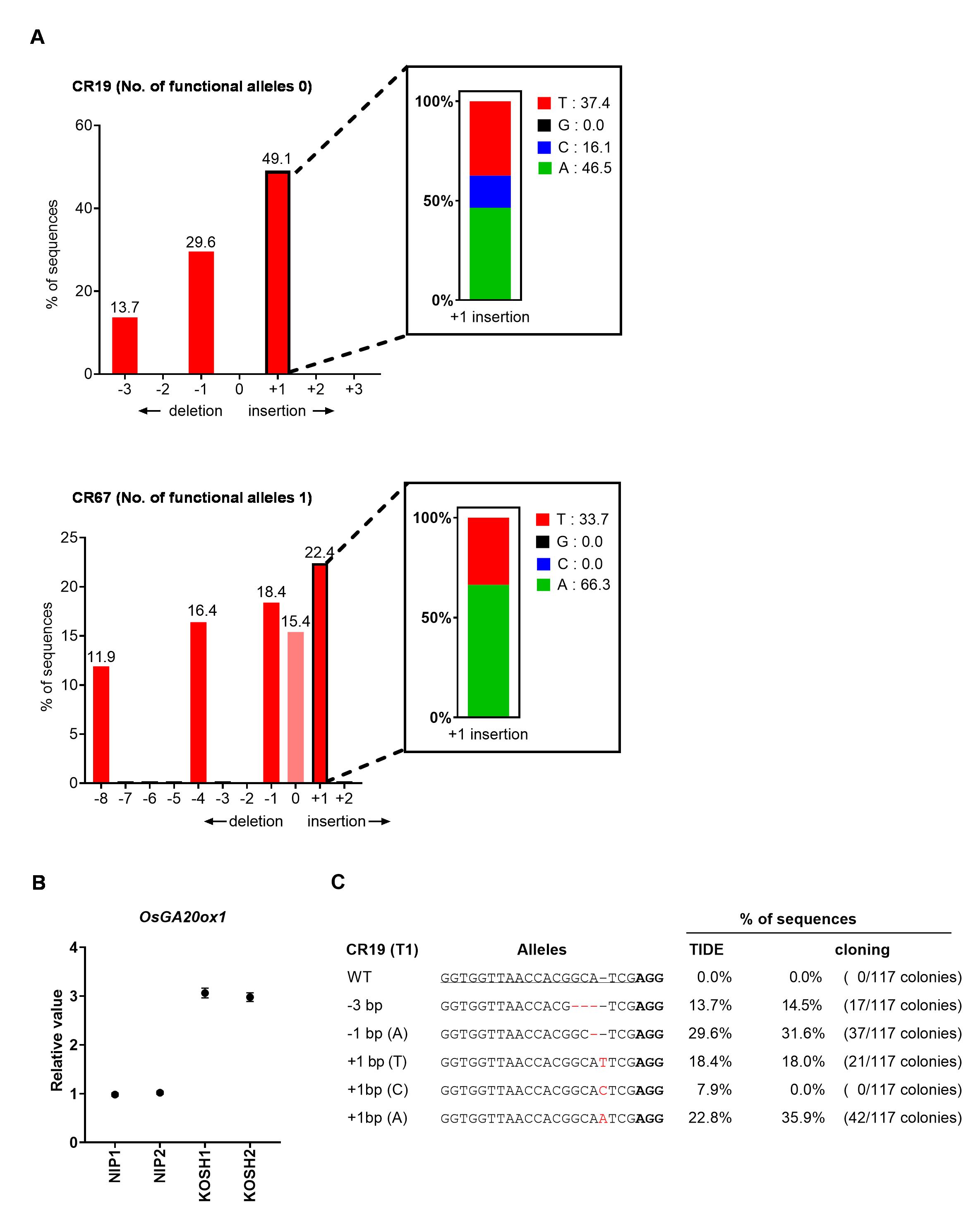

Supplement: Supplementary file 4 [file Image4.jpeg]

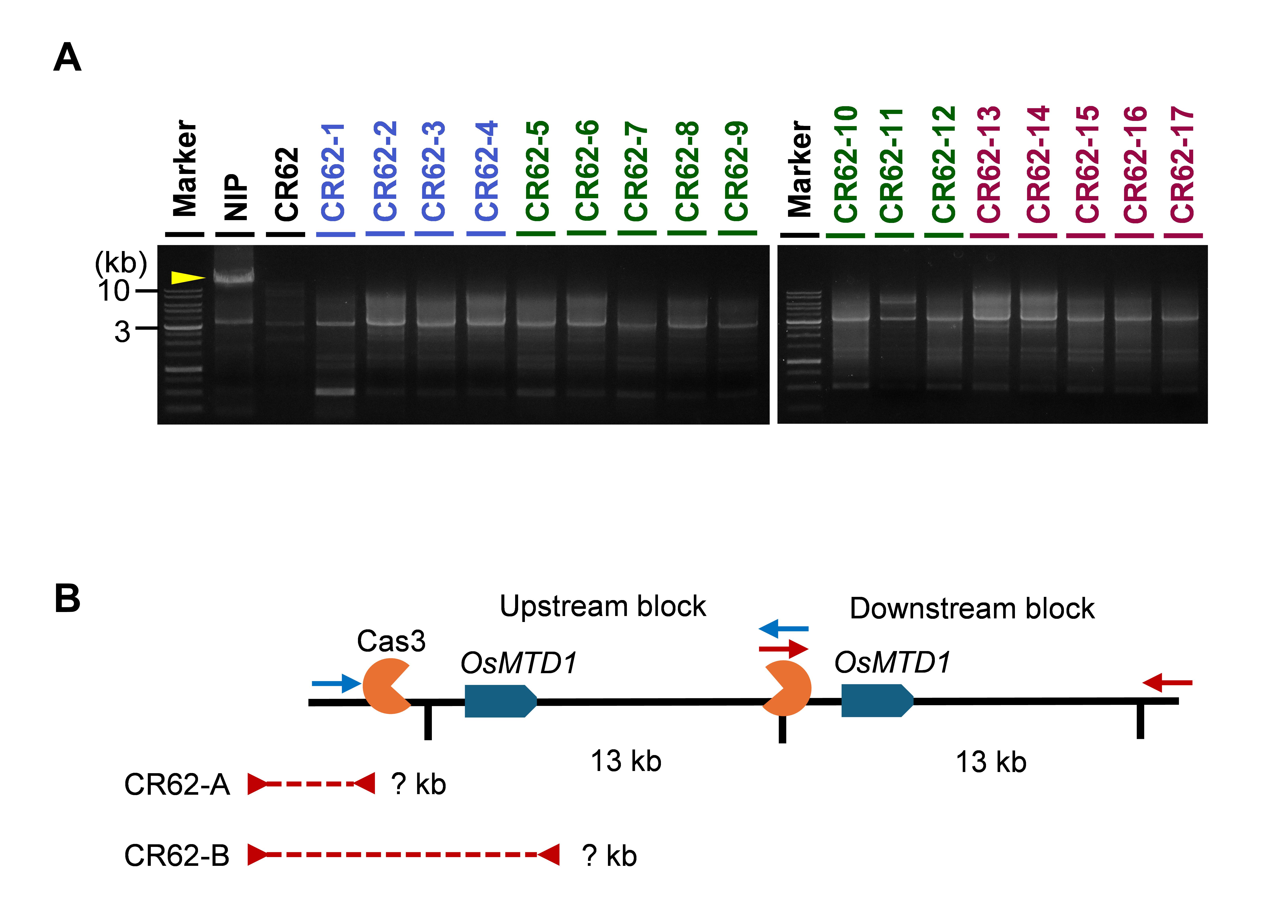

Supplement: Supplementary file 9 [file Image6.jpeg]
